# Advantages and challenges of metagenomic sequencing for the diagnosis of pulmonary infectious diseases

**DOI:** 10.1111/crj.13538

**Published:** 2022-09-06

**Authors:** Yan Chen, Li‐Chao Fan, Yan‐Hua Chai, Jin‐Fu Xu

**Affiliations:** ^1^ Department of Respiratory and Critical Care Medicine, Shanghai Pulmonary Hospital, Institute of Respiratory Medicine Tongji University School of Medicine Shanghai China

**Keywords:** application, metagenomic sequencing, pulmonary infections

## Abstract

**Objective:**

We aim to familiarize the application status of metagenomic sequencing in diagnosing pulmonary infections, to compare metagenomic sequencing with traditional diagnostic methods, to conclude the advantages and limitations of metagenomic sequencing, and to provide some advice for clinical practice and some inspiration for associated researches.

**Data Sources:**

The data were obtained from peer‐reviewed literature, white papers, and meeting reports.

**Results:**

This review focused on the applications of untargeted metagenomic sequencing in lungs infected by bacteria, viruses, fungi, chlamydia pneumoniae, *Mycoplasma pneumoniae* , parasites, and other pathogens. Compared with conventional diagnostic methods, metagenomic sequencing is better in detecting novel, rare, and unexpected pathogens and being applied in co‐infections. Meanwhile, it can also provide more comprehensive information about pathogens. However, metagenomic sequencing still has limitations. Also, the situations that should be applied in and how the results should be interpreted are discussed in this review.

**Conclusion:**

Metagenomic sequencing improves efficiency to identify pathogens compared with traditional diagnostic methods and can be applied in clinical diagnosis. However, the technology of metagenomic sequencing still needs to be improved. Also, clinicians should learn more about when to use metagenomic sequencing and how to interpret its results.

## Graphical abstract

Compared with conventional diagnostic methods, metagenomic sequencing is more sensitive and better in detecting novel, rare, and unexpected pathogens. Meanwhile, it can provide more comprehensive information about pathogens. However, many challenges exist when using metagenomic sequencing. By Figdraw (www.figdraw.com).

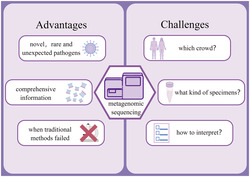

## 1 BACKGROUND

Pulmonary infections remain the leading infectious cause of morbidity and mortality worldwide.^1^ They can be caused by bacteria, viruses, fungi, chlamydia pneumoniae, *Mycoplasma pneumoniae*, parasites, and other pathogens. The conventional diagnostic progress is mainly based on the patients' clinical presentations, imaging manifestations, and laboratory examinations. The traditional laboratory tests to identify of pathogens are limited to serological assays, nucleic acid detection by specific polymerase chain reaction (PCR), and culture from respiratory samples (sputum, nasopharyngeal swabs, or tracheal aspirates).^2^, ^3^ As shown in Table 1, these approaches are classic but still have deficits. For instance, biomarker detection has low sensitivity, and culture processing is time‐consuming. The diagnostic yield can only reach 70%–80% even by using the most comprehensive diagnostic methods.^4^ A delay or misidentification of microorganisms directly exerts a significant effect on definitive antimicrobial therapy. The condition of patients deteriorates, and mortality increases if the causative pathogens are not identified timely. Thus, a new technology that can rapidly identify pathogens with high accuracy is desperately needed.

**TABLE 1:** Advantages and limitations of testing methods for diagnosing pulmonary infectious diseases

Diagnostic test	Advantages	Limitations
Serological assays	Potential in diagnosing acute infection	Low sensitivity during early infection
	Inexpensive	Low sensitivity in humoral immune deficiencies
PCR	High specificity and sensitivity in detecting viruses	Hypothesis‐depended
	Rapid	Requirement of prior sequence data for designing primers
	Inexpensive	Limited use in detecting unknown pathogens
Culture	Gold standard in diagnosis of pathogen	Low sensitivity after using antibiotics and antifungals
	Inexpensive	Limited use in testing fastidious organisms, unknown pathogens and virus
		Time consuming
Targeted NGS (e.g., 16S)	Capability in differentiating multiple species within one pathogen type	Limited use in detecting pathogens without currently available 16S sequence data
	High sensitivity	Requirement of prior sequence data for designing primers
		Difficulty in identifying pathogens to the species levels when existing a high degree of complete similarity across the length of 16S for some pathogens
		Expensive
Metagenomic NGS	Hypothesis‐free and unbiased	Difficulty in eliminating human host background
	High sensitivity	Hypersensitivity leading to false‐positive results
	Discovery of novel, rare or unexpected pathogens	Difficulty in interpreting the results for clinicians
	Capability in providing comprehensive information about pathogens	Expensive

Abbreviations: NGS, next‐generation sequencing; PCR, polymerase chain reaction.

Next‐generation sequencing (NGS), also known as high‐throughput sequencing or deep sequencing, is a deoxyribonucleic acid (DNA) sequencing technique. The release of the first massively parallel pyrosequencing platform marking the successful exploitation of NGS is in 2005.^5^ Compared with the gold standard for DNA sequencing, that is, Sanger sequencing, NGS has greatly improved the throughput, and the cost has dropped dramatically.^6^ The development of the technique NGS has made metagenomic sequencing a practicable approach to be applied in clinical diagnosis. Metagenomic tools can sequence all the nucleic acid from the host and pathogenic specimens. Different from targeted NGS approaches, such as 16S ribonucleic acid (RNA) gene amplification for bacteria,^7^ metagenomic sequencing is untargeted and can determine pathogens, regardless of their type, and provide sufficient information about pathogens, such as their antimicrobial genes and the human host response.^8^ Moreover, when aiming to use targeting antibiotics correctly, metagenomic sequencing can provide the needed results of culture and drug sensitivity reports within 24–48 h,^9^ a turn‐around time shorter than the turn‐around time of conventional diagnostic methods. Thus, metagenomic sequencing has the potential advantage than the traditional approaches in detecting the pathogen in infectious diseases.

Because the first report of metagenomic next‐generation sequencing to diagnose an infectious disease was reported, which identified a neuro‐invasive pathogen of cerebrospinal fluid,^10^ the applications of metagenomic sequencing in diagnosing infectious diseases such as sepsis,^11^ neurological infections,^12^, ^13^ and pneumonia^13^ gradually emerged. Here, we focus on the applications of metagenomic sequencing in lung infections, discuss its advantages and limitations compared with traditional diagnostic methods, and try to advise the applications of metagenomic sequencing in different situations.

## 2 APPLICATIONS OF METAGENOMIC SEQUENCING IN PULMONARY INFECTION

The metagenomic sequencing technique was introduced to clinical diagnosis and has such a short history that most research papers available are case reports with a few retrospective studies and even fewer prospective studies. The field of lung infections is no exception. Below here sketch the current application status of metagenomic sequencing in pulmonary infections.

In the last 5 years, the number of case reports using metagenomic sequencing to identify pathogens increased.^14^, ^15^, ^16^, ^17^, ^18^, ^19^, ^20^, ^21^, ^22^, ^23^, ^24^, ^25^, ^26^, ^27^ Among the 32 cases shown in Table 2, pathogens were identified in 30. Five of those 30 were bacteria, 20 were viruses, 3 were fungi, 1 was *Chlamydophila psittaci*, and 1 was *Spirometra erinaceieuropaei*. These results indicate that metagenomic sequencing is untargeted and can detect most types of pathogens. Clinicians prefer to use bronchoalveolar lavage fluid (BALF) (25/30) as a specimen, but sputum (2/30), throat swabs (2/30), blood (2/30), and pleural effusion (1/30) were also used.

**TABLE 2:** Clinical applications of mNGS in pulmonary infection disease

Ref.	Study	Case no	Specimen type	Pathogen identified	Confirmatory test of the metagenomic result
12	Nicole Fischer, 2014	1,2	BALF	Commensal bacteria	High‐throughput,16S rRNA sequencing, PCR, and serologic analysis
		3		*Chlamydophila psittaci*	
13	DagmaraW. Lewandowska, 2015	4	Throat swabs and stool samples	HEV‐C104	Whole nucleic acid high‐throughput sequencing
14	Kathryn M. Pendleton, 2017	5	Mini‐BALF	*Pseudomonas aeruginosa*	Culture, WGS, 16S rRNA sequencing
		6		*Staphylococcus aureus*	
15	Fugui Yan, 2017	7	BALF	HRV‐B91	Seroconversion of HRV‐B91 neutralizing antibodies
16	Yanpeng Li, 2018	8	Pulmonary secretions	HBoV1 and HRV‐C	PCR
17	Bailu Du, 2018	9	The mass at the root of the right thigh	*Spirometra erinaceieuropaei*	Serological tests using anti‐sparganum antibodies
18	Jian Wang, 2019	10	Sputum	GkV_CN‐GZ1	PCR
19	Bin‐Chan He, 2019	11	BALF	*Aspergillus fumigatus*	The serum G test and the BALF GM
		12			Sputum culture and the BALF GM
		13			The results of other tests are all negative
20	Yan Lin, 2019	14	Plasma	*Stenotrophomonas maltophilia*	/
21	Huahua Yi, 2020	15	Blood, sputum, and pleural effusion	*Legionella pneumophila*	PCR
22	Ancong Xu, 2020	16	Sputum and blood	*Acinetobacter baumannii*	Sputum culture
23	Liangjun Chen, 2020	17, 18	BALF	SARS‐Cov‐2	/
24	Roujian Lu, 2020	19–27	BALF or cultured virus or throat swab	SARS‐Cov‐2	PCR
25	Li‐Li Ren, 2020	28–32	BALF	SARS‐Cov‐2	Sanger sequencing and PCR

Abbreviations: BALF, bronchoalveolar lavage fluid; HEV, human enterovirus; HRV, human rhinoviruses; HBoV, human bocavirus; GkV, gemykibivirus; SARS‐CoV‐2, severe acute respiratory syndrome coronavirus 2; PCR, polymerase chain reaction.

Similarly, there is an increasing trend in the number of studies, which have included multiple samples since 2017.^28^, ^29^, ^30^, ^31^, ^32^, ^33^, ^34^, ^35^, ^36^, ^37^, ^38^ Among the 11 studies displayed in Table 3, four used children as the research subjects. BALF is obtained from an invasive operation. Other types of specimens, such as nasopharyngeal/oropharyngeal (NP/OP) swabs, sputum, blood, and endotracheal tube, are easier than BALF and safer to get. Thus, BALF is used less often. It is worth noting that a study published in 2018 used computed tomography (CT)‐guided puncture lung biopsy tissues.^31^

**TABLE 3:** Comparison of mNGS with traditional methods

Ref.	Study	Specimen type	Traditional tests	Potential clinical indications	No. of positive NGS results	No. of positive other methods' results	No. of cases tested
26	Jian Yang, 2011	NP aspirate samples	Conventional PCR and real‐time RT‐PCR	ALRTs	15	15	16
27	Xiaohui Zou, 2017	OP swabs	Real‐time PCR	Severe pneumonia	16	0	33
28	Robert Schlaberg, 2017	NP/OP swabs	Culture and PCR	CAP	13	0	70
29	Henan Li, 2018	lung biopsy tissues	Culture and smear	11 pulmonary infection, 3 pulmonary tuberculosis, 3 lung cancer, 3 pulmonary occupying lesions	15	12	20
30	Charles Langeliera, 2018	BALF	Culture, Aspergillus galactomannan assay, multiplex PCR, silver stain	CAP or HAP	13	6	22
31	Lauge Farnaes, 2019	Peripheral blood	Culture and PCR	CAP	13	6	15
32	Yun Xie, 2019	Sputum, blood and BALF	Conventional microbial tests	Severe pneumonia	27	23	48
33	Tingting Pan, 2019	BALF	Quantitative cultures, multiplex PCR	CAP	12	6	13
34	Yi Zhang, 2019	3 blood, 1 tissue sample, 4 sputum and 7 BALF	Smear and culture	PCP	15	6	15
35	Matt S. Zinter, 2019	33 BAL, 4 mini‐BAL, 4 ETA	Conventional laboratory methods	Pulmonary infection	25	17	41
36	Libing Yang, 2019	ETA	Culture	VAP	10	9	14

Abbreviations: ALRTs, acute lower respiratory tract infections; CAP, community‐acquired pneumonia; ETA, endotracheal aspirate; HAP, hospital‐acquired pneumonia; NP, nasopharyngeal; OP, oropharyngeal; PCP, pneumocystis pneumonia; PCR, polymerase chain reaction; RMPP, refractory *mycoplasma pneumoniae* pneumonia; VAP, ventilator‐associated pneumonia.

## 3 DIAGNOSTIC YIELD

In studies targeted to identify all types of pathogens, metagenomic sequencing showed a diagnostic yield (number of positive results/number of cases tested) between 56.25% and 92.31%, whereas the conventional microbiologic tests showed 27.27%–64.29%.^31^, ^32^, ^33^, ^34^, ^35^, ^36^, ^37^, ^38^ In all studies, metagenomic sequencing detected pathogens at least as well as the conventional tests,^28^, ^29^, ^30^, ^31^, ^32^, ^33^, ^34^, ^35^, ^36^, ^37^, ^38^ even compared with PCR which is famous for its high specificity and sensitivity in detecting viruses.^28^ Furthermore, metagenomic sequencing could produce positive results in specimens previously considered by the traditional laboratory tests to be negative.^29^, ^30^ However, it is worth noting that assay hypersensitivity may lead to false‐positive results.

As for the capability of metagenomic sequencing in detecting different types of pathogens, a retrospective observational study conducted between 2010 and 2018 in China revealed the pathogenic bacteria positive rate of metagenomic sequencing was higher than that of traditional methods (68.7% vs. 45.4%, *p* = 0.006, Pearson *χ* ^2^). Meanwhile, there was no significant difference between the two methods on the pathogenic fungal positive rates.^34^ However, another study showed the advantages of metagenomic sequencing to detect fungi, viruses, and opportunistic pathogens in immunocompromised hosts, whereas no significant difference existed in bacterial detection between the two techniques.^35^ In another recent study, the detection rate of *Pneumocystis* pneumonia (*PCP*) was higher in high‐throughput sequencing compared with conventional methods such as Wright–Giemsa stained smear and microscopy identification. The conventional methods failed to detect *Pneumocystis jirovecii* in 8 of 13 samples, whereas metagenomic sequencing was successful.^35^ The type pathogen that can best be detected by metagenomic sequencing is still inconclusive. More research is needed for helping clinicians achieve a better judgment in interpreting inconsistent results of metagenomic sequencing and other methods.

## 4 THE ADVANTAGES OF METAGENOMIC SEQUENCING

### 4.1 Metagenomic sequencing can detect novel, rare, and unexpected pathogens

Metagenomic sequencing is a target‐independent approach that offers a more comprehensive view of the pathogenic agents and provides a detection of common and unexpected pathogens in samples.^39^ This technology does especially well in detecting rare pathogens. *Chlamydophila psittaci* is excluded from in standard diagnostic panels because of its rarity. However, metagenomic sequencing identified *Chlamydophila psittaci* successfully in a patient who had severe *chlamydial* pneumonia with acute respiratory distress syndrome (ARDS) in a case reported in 2014.^14^ The gold standard for diagnosing a parasitic disease is to find the parasite. A rare case was reported in which metagenomic sequencing diagnosed an 11‐year‐old patient presented with pulmonary and pericardial effusion. The genomic DNA of *Spirometra erinaceieuropaei* was identified in a lump derived from the root of the right thigh, while *sparganum* was not detected in the dissected tissues.^19^ Metagenomic sequencing played a decisive role in achieving a definitive diagnosis in this case. Similarly, a case reported in 2017 identified human rhinoviruses B91 (HRV‐B91), a rarely reported subtype, in BALF by deep sequencing. Here, conventional real‐time PCR assay, a conventional approach for viral identification due to its high specificity and sensitivity, produced negative results.^17^ The reason for which this molecular method did not identify HRV‐B91 successfully is its target specific, which may lead to the missing of the novel, rare, and unexpected viruses.

Another merit of metagenomic sequencing is to detect a novel viral strain. A case reported in 2019 was the first to detect the *Gemykibivirus* (GkV) genome using metagenomic sequencing from the sputum of a patient with pneumonia. It was the first time that the GkV was found in the respiratory tract.^20^ Recently, the novel coronavirus, severe acute respiratory syndrome coronavirus 2 (SARS‐Cov‐2), which caused severe pneumonia in Wuhan, China, was sequenced by metagenomic sequencing.^25^, ^26^, ^27^ Deep sequencing methods provided a great contribution in raising our awareness of a novel viral strain of this virus.

### 4.2 Metagenomic sequencing is more sensitive than traditional methods

Culturing is a conventional and widely used method to identify pathogens. However, culturing is time‐consuming and many pathogens that need specific culture conditions are difficult to grow. *Legionella pneumophila* needs a specific medium to be cultured; thus, it is harder to obtain the correct diagnosis. Metagenomic sequencing detects pathogens regardless of their taxonomy. It can identify pathogens that do not grow in culture. In a recent report, *Legionella pneumophila* was detected in all blood, sputum, and pleural effusion samples by metagenomic sequencing taken from a patient with community‐acquired pneumonia.^23^ Many studies have revealed that metagenomic sequencing could identify more types of pathogens than the culture method.^40^, ^41^, ^42^

Metagenomic sequencing also plays an essential role in the identification of pathogens in pulmonary fungal infections. Except for smear by microscopy, pathogen culture and PCR, serum or BALF galactomannan (GM) test, and serum 1,3‐β‐d‐glucan (G) tests help diagnose invasive pulmonary *aspergillosis* (IPA).^43^ However, problems such as time–cost and low‐yield production still exist in traditional methods, making it difficult to diagnose IPA. In case 13, metagenomic sequencing was the only approach to detect the *Aspergillus fumigatus*, whereas other conventional methods failed.^21^ Clinicians can consider metagenomic sequencing as a complementary method to diagnose IPA.

### 4.3 Metagenomic sequencing is suitable to detect co‐infections

Metagenomic sequencing is untargeted. The capability to identify bacteria, viruses, fungi, parasites, and other pathogens at the same time in only one approach makes metagenomic sequencing widely appealing for co‐infection cases. A rare case reported in 2018 was an adult co‐infected with both human rhinovirus (HRV) and bocavirus (HBoV).^18^ Almost every study covering multiple objects included samples in which a mix‐infection was detected.^28^, ^29^, ^30^, ^31^, ^32^, ^33^, ^35^, ^36^ Metagenomic sequencing can rule out co‐infections as well. A study published in 2018 reviewed 675 patients with childhood refractory *Mycoplasma pneumoniae* pneumonia. The culture results revealed that 18 of the 675 specimens were positive for bacteria. Another 18 specimens were picked out randomly from the remained 657 specimens to be tested by metagenomic sequencing again. Researchers used the metagenomic sequencing as a validation to assure the culture results.^44^

### 4.4 Metagenomic sequencing provides comprehensive information about pathogens

Metagenomic sequencing allows the evaluation of phylogenetic analysis, strain‐level typing, and antigenic epitope characterization of pathogenic genomes. Phylogenetic analysis, based on sequence alignment, helps to manage endemics and outbreaks by finding the origins of viruses. For example, SARS‐CoV‐2 was classified into the genus β‐coronavirus, subgenus Sarbecovirus, and its sequence was shown to be closest with bat SARS‐like coronavirus (SL‐CoV) ZXC21 (76.5%–91.2%) and bat SL‐CoV ZC45 (76.9%–91.2%) by phylogenetic analysis.^25^, ^26^, ^27^ The fact that SARS‐CoV‐2 probably originated from the bat was amply reported. Notably, the genetic similarity between the SARS‐CoV‐2 strains and SARS‐CoV (79%–82%) or the Middle East respiratory syndrome (MERS)‐CoV (50%–51.8%) offers a direction for managing the Corona Virus Disease 2019 (COVID‐19).^25^, ^26^, ^27^

Metagenomic sequencing can discover novel antibiotic resistance genes in pathogens and investigate the antibiotic resistome of new strains.^45^, ^46^ For instance, 18 possible antibiotic resistance genes were identified from the *Acinetobacter baumannii* strain DMS06669, isolated from the sputum of a patient with hospital‐acquired pneumonia. Notably, 8 of 18 genes appeared in *A. baumannii* for the first time.^47^ Based on the information in the literature, relevant databases were established. The Comprehensive Antibiotic Resistance Database (CARD; http://arpcard.mcmaster.ca) is a good example, and it renders the service for predicting an antibiotic resistance phenotype from a genome sequence.^48^, ^49^, ^50^

### 4.5 Metagenomic sequencing shortens the turnaround time

Currently, the burden of multidrug‐resistant bacteria is getting heavier. We know that prolonged empirical antibiotic therapies may cause the emergence of drug‐resistant bacteria such as *Achromobacter xylosoxidans* and *Stenotrophomonas maltophilia*.^51^, ^52^ In numerous cases, the failure of recognizing resistant bacteria has been reported to delay the providing of appropriate antibiotic treatment for patients, resulting in severe clinical consequences.^53^, ^54^ Therefore, timely, accurate, and targeted antibiotic treatment is important. A study published in 2019 provided evidence that metagenomic sequencing reduced the mortality of severe pneumonia to 16.67% compared with 42.3% in control.^34^ The main reason may be that using metagenomic sequencing (average of ~48 h) is faster than conventional tests (average of 3–5 days) in achieving accurate results of culture and drug sensitivity reports when a bioinformatics personnel is available.^9^

As an emerging metagenomic sequencing technology, nanopore sequencing takes less time to generate the sequence. A comprehensive case report published in 2017 identified *Pseudomonas aeruginosa* and *Staphylococcus aureus* using real‐time metagenomics in mini‐BALF of two patients with bacterial pneumonia. The first application of the MinION (Oxford Nanopore Technologies, Oxford, UK), a new‐to‐market palm‐sized DNA sequencer, to identify pathogens in pneumonia revealed that real‐time metagenomics is quicker and much more convenient than culturing. It took 9 h to get the sequence from one patient's specimen, which was well aligned with the *Pseudomonas aeruginosa* sequence.^16^ Another recent study was the first to detect pathogenic fungi using nanopore sequencing with a workflow of 10 h, and the result was consistent with routine diagnostic methods. Patients in this study were diagnosed with *PCP*, and nanopore sequencing confirmed the diagnosis.^55^ Nanopore sequencing has top speed among all metagenomic sequencing technologies.^56^

## 5 THE CHALLENGES IN THE APPLICATIONS OF METAGENOMIC SEQUENCING FOR CLINICIANS

### 5.1 The scenarios suitable for applying metagenomic sequencing

Metagenomic sequencing is good at identifying rare or novel pathogens, and it also does well in multi‐infections. To our knowledge, patients with an immune deficiency can be easily infected by opportunistic pathogens or with multiple infections. Thus, metagenomic sequencing is particularly suitable for those patients who are immunocompromised or immunodeficient. Under these conditions, when faced with infants, children, aged people with basic diseases, and repeatedly hospitalized people, clinicians could apply metagenomic sequencing to detect pathogens as soon as possible. Also, as an untargeted and comprehensive detecting method, metagenomic sequencing can be used in people suspected of being infected by specific pathogens, people with unexplained infections, people who cannot be diagnosed by repeated traditional microbial detection technology, or people who are critically ill.^16^, ^57^, ^58^

### 5.2 The selection of specimens

Appropriate selection of specimens helps improve the accuracy of diagnosis. A study published in 2012 investigated the complexity and distribution of microbiota in chronic obstructive pulmonary disease (COPD) patients using a 16S ribonucleic acid (RNA) gene‐based technique to analyze sputum, bronchial aspirate, BALF, and bronchial mucosa. It revealed that the outcomes of the former two samples were similar, whereas those of the latter two were similar. This study indicated that the two most used clinical samples—sputum and BALF—were different. Sputum represented the upper bronchial tree samples, while the BALF represented the lower bronchial tree samples.^7^ This study indicated that BALF was a better specimen to diagnose lung infections. The accomplishment of strict negative controls and protected bronchoscopic sampling techniques including using a catheter with a wax‐sealed tip alleviated a risk for contamination with pharyngeal microbiota when a bronchoscope went through the upper respiratory tract.^59^ However, some patients may refuse invasive operation or cannot withstand the bronchoscope examination. Another study published in 2019 focused on comparing two sampling approaches (tracheal aspirate and mini‐bronchoalveolar lavage) in patients with pneumonia who were critically ill. Metagenomic sequencing was used to analyze the abundance of microbiota taken by two types of sampling, and the result showed no significant difference. This study challenged the old idea that less invasive tracheal aspirate sampling was inferior to mini‐bronchoalveolar lavage sampling because of the potential contamination from oropharyngeal microbiota. The same study demonstrated that the less invasive tracheal aspirate could replace mini‐bronchoalveolar lavage.^60^ DNA sequencing of cell‐free plasma (CFPDNA) is a new commercial method that only requires blood to test, which means it is another noninvasive approach. It has a satisfactory detection rate for bacteria and fungi. However, CFPDNA sequencing has limitations in detecting RNA viruses.^33^, ^61^, ^62^ Also, a study suggested that lung biopsy tissues also had the potential to identify pathogens. Thus, metagenomic sequencing displays many advantages in speed and sensitivity.^31^ However, obtaining CT‐guided puncture lung biopsy tissues harm the patient.

In a nutshell, when selecting specimens, the patient's will, physical condition, and representativeness should all be taken into consideration.

### 5.3 The interpretation of metagenomic sequencing results

The detection of microorganisms by metagenomic sequencing can prove their existence but not their pathogenicity because it is difficult to distinguish normal microbiota, colonized microbiota, contamination, or bona fide pathogens.^8^ The lung is considered to be sterile, but this view has been overturned nowadays. Microbial communities exist in the lungs of healthy humans.^63^ Also, the composition of lung microbiota in those affected with chronic pulmonary diseases differs from healthy people.^64^, ^65^, ^66^ Nucleic acid contamination may occur in several procedures during the workflow. As for clinicians, they should be prudent to avoid contamination when collecting specimens. Notably, in theory, contamination with commensal oral flora organisms seems to be inescapable.^67^

Without a standardized protocol, it is difficult for clinicians to interpret the results of metagenomic sequencing. Databases that consist of potentially contaminating genera help clinicians recognize normal microbiota, contamination, and bona fide pathogens.^68^, ^69^ Negative controls that monitor external contamination are recommended to be sequenced simultaneously.^56^, ^68^ Quantitative and semiquantitative statistical analyses have been reported to distinguish colonization from infection.^16^, ^32^ However, an acknowledged approach is required. Clinicians should also concentrate on the clinical manifestations of patients and outcomes of other traditional diagnostic methods. For instance, whether opportunistic pathogens caused disease depends on the patients' presentation.^56^ Remarkably, the lung pathogenicity of a microbe identified by metagenomic sequencing should have been evidenced by the existing literature.^32^ Furthermore, negative metagenomic sequencing outcomes may be due to very low quantities for sequencing^42^ and do not exclude the existence of causative agents. In summary, clinicians should be well‐rounded when interpreting metagenomic sequencing reports. Cooperation within a group consisting of experts from medical microbiology, computational biology, and clinicians to interpret results of metagenomic sequencing is advised.^9^

## 6 CONCLUSION

The mortality associated with pulmonary infections is higher than that of other infectious diseases.^1^ Up to 60% of pulmonary infection cases were treated without gathering evidence for the presence of pathogens,^4^, ^13^ as traditional clinical diagnostic methods have low sensitivity and are time‐consuming. It is important to identify pathogens as early as possible because this information promotes the management of diseases and consequently improves the prognosis of the patients.

NGS has become a research hotspot in recent years. However, this technology still needs improvement to narrow the gap between academic research and clinical applications. Importantly, several obstacles should be overcome to improve specificity of the technology. The first is to isolate pathogens from complicated clinical samples reliably. Human sequences need to be removed as much as possible. A study compared three DNA extraction methods by eliminating the nucleic acid of hosts and found that the one with Bensonase could obtain a higher yield of microbial DNA.^70^ Reference sequence databases should be improved by verifying the accuracy of the sequences and the databases should also be updated periodically. The specification operation of clinicians to collect specimens which can avoid contamination is also an approach to improve the specificity. On the other hand, the cost should be cut down because it is still much higher than that of routine diagnostic methods.^71^

As for the applications of metagenomic sequencing in lung infections, many challenges exist. When a patient has a pulmonary infection, clinical samples are commonly taken from sputum, NP/OP swabs, BALF, and lung puncture biopsy tissues. The location, shape, and size of lung lesions along with sputum quality and invasive operation tolerance of patients should be in consideration to determine which type of sample is the most appropriate for detecting pathogens in lung infections using metagenomic sequencing. Commonly, BALF is the first choice as it is taken from lower respiratory tract directly. Sputum expectoration and induced sputum can be alternative if patients reject invasive fiberoptic bronchoscopy. Patients with viral pneumonia usually do not have obvious expectoration. For those suspected of viral lower respiratory tract infection, NP/OP swabs can be collected. For peripheral pulmonary lesions, localized lesions, or mediastinal/hilar lymphadenopathy, invasive operation should be selected individually according to the characteristics of the lesions to obtain tissues for examination. Another challenge is to interpret the results. Clinicians should combine results from all tests with patients' manifestations and interpret them from all possible angles. Whether microorganisms detected are pathogenic bacteria, colonizing bacteria or background bacteria should be carefully discriminated. And the detection results should be cross verified by other traditional microbial technologies.

In summary, metagenomic sequencing is a diagnostic technology that complements current diagnostic methods for pathogen detection in pulmonary infections. In addition to the diagnostic role, metagenomic sequencing also informs genotyping, predicting drug resistance, and guiding the medication. This technology still needs to be improved, and clinicians must enhance their understanding of its results. Metagenomic sequencing will be an important supplement for the targeted diagnostic methods for pathogen detection.

## CONFLICTS OF INTEREST

The authors declare that they have no conflicts of interest with the contents of the article.

## ETHICS STATEMENT

No ethics approval was needed, no human subjects were involved in this review paper, and no consent to participate and publish was needed.

## AUTHOR CONTRIBUTIONS

YC and JFX were the major contributors to the writing of the manuscript. LCF and YHC revised the manuscript. All authors read and approved the final manuscript.

## Data Availability

Data sharing is not applicable to this article as no new data were created or analyzed in this study.
